# Domestic

**Published:** 1840-12-05

**Authors:** 


### DOMESTIC.

### NEW YORK HOSPITAL.

*Case of Injury of the head, paralysis of the left side; coma. Death on the ninth day. Laceration of the brain, extravasation in the centre of the right hemisphere. Pneumonia.*—Robert Kerr, a native of Scotland, aged 30, was admitted moribund, January 5th, 1840, and died in ten hours after admission. Those who brought him to the hospital stated that while at sea, eight days before admission, he had fallen from a yard-arm, and that he had remained insensible ever since, with total loss of power of the whole of the left side. He was examined five hours after death.

Externally he was marked by a bruise over the left eye, by a second on the right thigh, and a third and more extensive one over the left hip. The abdominal viscera were healthy, with the exception of the liver, which was of its natural colour, but much engorged with blood. Both lungs were congested and adhering by strong but recent attachments at their upper portion to the parietes of the chest. No fracture was observed in the skull; yet the cerebral substance appeared to have been lacerated, and about half an ounce of congealed blood was found at the seat of laceration in the central portion of the middle lobe on the right side.

*Case of Injury of the head with laceration of the scalp; coma approaching gradually. Death in six hours. Extravasation from rupture of a small vein on the surface of the brain.*—Joseph McMahon, a native of Ireland, aged 28, a carpenter, was admitted late at night on the 8th of June, apparently intoxicated, but able to give a confused account of himself. He had recently been injured probably by a fall. A very small lacerated wound was observed upon his scalp. This was dressed by the house-surgeon, and the patient thus left for the night. The inmates of the ward stated that he appeared soon afterwards to fall asleep, and to breathe heavily. He was found dead at the early visit on the following morning.

No fracture could be discovered in the skull, although carefully examined. About eight ounces of blood had been extravasated, forming a layer of coagulum under the serous envelopes on the surface of the right hemisphere, and reaching to the base of the brain. The blood had escaped from an evident rupture of one of the small veins, ramifying between the convolutions on the upper and central portion of the right hemisphere.

*Case of Apoplexy. Death within ten hours of the attack. Disorganization of the cerebral substance at the centre and base of the brain; extensive extravasation filling the ventricles.*—John E. Williams, of Pennsylvania, a boatman, aged 23, was admitted in a state of insensibility, towards evening, June 26th. A few hours previous to admission, while standing in the street, he had been suddenly seized with an apoplectic fit, for which he had been bled without relief. Counter irritants and revulsives were employed after his admission; and the house-surgeon again bled him in the evening. He continued to sink, however, and died towards midnight. He was examined about twelve hours after death.

The configuration of the body was that of an athletic man, moderately fleshy, and well developed. The left ventricle of the brain was distended with a clot of blood, which on the lower surface appeared gradually to mix with the softened and disorganized cerebral substance, that forms the floor of the ventricle. The right ventricle was also distended with blood, but the walls of this cavity were apparently healthy. The coagulated and grumous blood filled, also, the third and fourth ventricles. The substance of the brain around the tubercula, quadrigemina, upper and posterior portion of the medulla oblongata, and upper anterior portion of the cerebellum, was softened and disorganized, and in places intermixed with grumous blood. The medullary mass, where not thus broken down, was even less vascular than natural, and of a remarkably hard and tough consistence. The membranes of the brain were unusually dry.

*Remarks.*—It was subsequently ascertained that this young man, though not subject to intoxication, was habituated to high living, and that not long before his death he was known to have had one or two epileptic fits. On the morning of the day upon which he was seized with apoplexy, he appeared to be in perfect health. During the day, which was excessively warm, he had exerted himself in preparing his boat for a pleasure excursion.

*Case of Fracture and depression of the sternum; injury of the back; general paralysis. Death in* 48 *hours. Extensive laceration of the pericardium; contusion of the heart; laceration of the right lung; fracture of one of the ribs; fracture of the fifth cervical vertebra.*—Ira Boarman, of Massachusetts, a seaman, aged 36, was admitted at noon on the 25th of March, having about two hours before admission, while standing near the hatchway of a ship, been struck on the chest by a bundle of hay, and precipitated into the hold, falling about twenty feet, and striking with his back against some bars of iron.

At the time of admission he possessed neither sensation nor power of motion in any part of his body below the diaphragm; the upper extremities were paralyzed, but retained their sensibility in all parts above the elbows; and soon after admission he began to complain of severe pains in his arms. His breathing was somewhat impeded, but his voice was strong and clear; his pulse was apparently natural at first, but afterwards became intermitting and irregular; the temperature of the body was about that of health. He could only lie flat upon his back; the slightest movement of his head or body gave him excruciating pain, and consequently it was difficult to determine the exact seat of injury on his back. The integuments over the sternum were not broken; there was some infiltration beneath them, and the bone itself was depressed.

He continued in this condition, breathing with tolerable freedom, and when left quiet, complaining of but little pain during the rest of the day. On the following day his bowels became tympanitic, he was unable to void his urine or fæces; his respiration more embarrassed, and his pulse intermitting. His urine was drawn off by the catheter, and his bowels moved by a stimulating enema. He sunk very gradually, and died about forty-eight hours after the accident.

The cellular tissue over the sternum was injected with blood; a comminuted fracture of the sternum, with depression, was detected between the second and third ribs. The anterior portion of the pericardium was lacerated longitudinally throughout almost its whole extent. A contusion, apparently with a superficial abrasion, about as large as half a dollar, was observed in the anterior wall of the right ventricle of the heart itself. Considerable ecchymosis existed under the serous surface of the pericardium, but no blood was found in its cavity. The lower lobes of both lungs were much engorged with blood. There was a laceration about an inch and a half long in the posterior part of the upper lobe of the right lung, caused by fracture and depression of the third rib at that point. The cavities of the pleura were free from blood. A fracture was found extending through the body of the fifth cervical vertebra.

*Remarks.*—The extensive injury of the pericardium, and contusion of the walls of the heart in this case, were not anticipated before the examination: the state of the circulation, and the patient's general symptoms, gave but very equivocal evidence of these injuries.

In the following case of fracture of the sternum, without very evident injury of the heart, the patient died almost immediately after the accident.

*Case of Fracture and depression of the sternum; compound fracture of the leg. Death in*

*about one hour. Extravasation into the cavity of the pleura; laceration of the liver.*—Thomas Matthews, an Irish seaman, aged 25, was admitted on the 23d of May, having recently been precipitated from the topmast of a schooner, striking as he descended against the bulwarks of the vessel, and falling into the river. When admitted he was wet and cold, and no pulse could be detected at the wrist. He was sensible, however, and requested to be turned on his side, as it gave him great pain to lie on his back. No reaction followed, and he died in about an hour after the accident.

He had suffered a compound fracture of the right leg; the sternum was fractured at its upper third, and the lower portion had been driven inward so as to lacerate the pleura. The cavity of the pleura contained a great quantity of blood. The left lobe of the liver was lacerated.

*Case of Tumour on the chest, connected with the caries of one of the ribs. Erysipelas, pleuro-pneumonia, and pericarditis supervening in the progress of the case, and leading to its fatal termination.*—John McClaron, an Irishman, aged 42, seaman, of good constitution and temperate habits, was admitted on the 14th of February, on account of a swelling that had existed several months on the left side of his chest, and that had of late given him much pain, and prevented him from attending to his occupation.

A flattened tumour scarcely elevated enough to be at first glance perceptible, existed on the left lower and posterior portion of the chest near the angle of the ribs. By careful pressure over the centre of this, a small elastic and circumscribed swelling, about an inch in diameter, flatttened, and apparently attached to the sixth rib, was detected; and one or two smaller swellings of similar character were afterwards discovered in the neighbourhood of this, all apparently connected with the rib. The bulging of the integuments caused by these small but deep tumours, gave to the surface the appearance of a flattened tumour, nearly circular, and about three inches in diameter. Pressure over this gave the patient no uneasiness. No evidences existed of any disease within the thorax. He could assign no specific cause for the swelling, but thought he might have formerly injured his side by a fall.

About the 1st of March, a puncture was made with a lancet through the swelling into the largest of the elastic tumours which appeared to be attached to the rib. Slight hæmorrhage, and trifling discharge of concrete matter, followed the puncture. The tissues above the rib were found to be thickened and consolidated. In a few days the wound began to suppurate, and the patient being now free from pain, left the hospital. The discharge, however, becoming gradually more abundant, he returned in ten or twelve days. The opening was now dilated, no diseased bone could be detected; the sore was treated as a fistulous ulcer.

On the 22d of April, several small fragments of bone were found loose in the bottom of the fistula. The integuments were again divided so that the finger could be introduced, and the whole of the detached pieces, some of them nearly as large as peas, were carefully removed. The patient's general health was still very good, and in the course of a few days the wound had almost entirely closed.

May the 7th, having on the previous night been fatigued by setting up with a very sick patient, and having changed his room for a smaller one, in which he was exposed to the draught of a window, he was seized towards midnight with a severe chill; and on the following morning he was found with considerable pain. In the course of the day he was bled freely, took some cathartic medicine, and was put on the use of spiritus Mindereri. As he complained of pain in his side, the chest was examined, but gave no evidence of any marked disorder.

May 9th. Febrile excitement somewhat diminished; pain in the left side still continuing. Cups were applied to the chest, and the patient put on nauseating doses of Tart. Ant. dissolved in spts. of Minderer, to be repeated every two hours.

May 10th. Up to this date the symptoms were apparently indicative of pleuritis; but this morning the integuments over the left side of the chest were in a state of acute erysipelatous inflammation. He still complained of pain in the side, and now over the scrobiculus cordis, and of pains in other parts of his body. The spts. Mindereri and antimony were continued, and the inflamed surface was anointed with mercurial ointment.

May 11th. The general excitement still continuing, and his bowels being somewhat constipated, he was put on the following mixture. ℞ Sulph. Magnes. ℥i. Tart. Ant. grs. viii. Aq. pluvial. ℥vij. Of this he bore without producing nausea, ℥ss. every two hours.

May 12th. This morning his general expression was haggard, his skin was bathed in perspiration, his pulse frequent and small; he had passed a restless night, and the pain in the chest was still severe. But much of this was attributed to the erysipelatous inflammation. His bowels still remaining constipated, he was directed Pulv. Purgans. ʒiss. Again cupped on the anterior part of the chest, and the antimonial solution continued. In the afternoon, the cathartic medicine not having as yet operated, a stimulating enema was administered which produced a small evacuation of fœcal matter.

May 13th. Pulse full and less frequent, skin moist, pain abated, erysipelas subsiding. The cathartic medicine has produced no further action of the bowels. Suspend the antimonial solution, and have Sub. Mur. Hydrarg. grs. xx.

May 14th. Symptoms as yesterday. Bowels moved by an enema. In the evening he had an anodyne of Solut. Sulph. Morph. gtt. xx.

May 15th. Symptoms as before. Resume the spts. of Mindereri without the antimony. The little food that he had hitherto taken since the febrile attack, had been of the simplest kind. This afternoon his pulse was for the first time observed to intermit. Towards night he evidently grew worse; his voice became weak and feeble; his respiration hurried; his skin purplish, cool, and bathed in clammy perspiration. It was necessary to suspend the diaphoretic, and administer small portions of brandy toddy, made weak, which he continued to take through the night.

May 16th. Reaction again established; skin warm and dry; pulse full but intermitting. In the afternoon he was again threatened with approaching collapse. His bowels were moved by a stimulating enema, and during the night it was found necessary to resort again to the brandy.

May 17th. The patient evidently sinking; the erysipelas had entirely disappeared; but the whole of the integuments on the posterior part of the body were injected as if by the gravitation of venous blood, presenting an appearance precisely similar to that produced by stasis of blood in bodies that have been for some days dead. The inflammation on the surface had for several days prevented any attempt at exploration of the chest. But this morning the decrepitating sound was detected in the lower lobe of the left lung. He died about five o'clock, P. M.

*Post-mortem examination* nineteen hours after death. The pericardium was distended with serum, and its inner surface coated on all parts with a layer of plastic lymph, about a quarter of an inch thick, extended as well over the heart as over the free portion of the serous surface. The substance of the heart was in other respects healthy. The left lung was firmly united to the point of the chest opposite the diseased portion of the rib. The remaining portion of the pleura was free from adhesions, but it was coated like the pericardium with a recent deposit of plastic lymph that adhered to it very slightly. The cavity of the pleura contained about a pint of serum. The lower lobe of the left lung was in a state of red hepatization; the upper lobe was free from disease. The other viscera were to all appearance healthy.

The diseased rib near its angle was somewhat thickened, and rather broader than natural; and contained a deep notch on its upper edge large enough to admit the end of the finger, and extending rather more than half way towards the lower edge of the bone. The lower edge was rough and irregular, and was also marked by a smaller notch. The rib on the sternal side of these was also perforated by a hole on its outer surface. These irregularities gave the bone the appearance of having once been fractured and afterwards united by callus. The dead fragments that had been removed through the opening in the integuments, had probably been exfoliated from the spots now marked by the deep notches. These notches were prevented from communicating with the cavity of the pleura, both by the periosteum, which was thickened and of a cartilaginous hardness, and by the firm adhesion between the lung and the side of the chest. This adhesion was evidently of ancient date.

*Remarks.*—This patient had not had the least cough during the whole of his disease, and his respiration was free from embarrassment until within a few days of his death. The disturbance in the circulation, the irregularity of the pulse, and the sudden sinking, with the severe pain over the region of the heart, gave at this late period the first suspicions of the inflammation of the pericardium which was detected after death. Had the extensive disease within the chest been evident at first, it would have been met by more active depletion; but it had been almost completely masked by the accompanying erysipelas. And it is worthy of remark that this superficial inflammation was as near as possible coextensive with the inflammation on the inner surface of the chest.—*Dr. Watson's Reports in New York Journal of Med. and Surg.*

---

*Case of Aneurism of the Aorta.*—James M'Guinnative, of Ireland, aged about twenty-six years—a stout muscular man, about five feet ten inches high—a stage driver by occupation, and of intemperate habits, was admitted July 2d. Stated that he had always been healthy up to last winter—that in January last, from exposure incident to his occupation, he took a severe cold, attended with cough and pain in one side, could not recollect which—that he was puked, purged, &c. and that he got better, but not entirely well. That he was able to go about in a short time, but continued to have a dry cough, attended with some dyspnœa, &c. which prevented him from resuming his occupation. That this state of things continued through the remainder of the winter and spring, and that he began to get worse about two or three weeks before he entered the hospital.

*Symptoms when admitted.*—*Surface*—skin in good condition, inconsiderable emaciation.—*Head*, normal.—*Thorax*—cough troublesome, occasional slight dyspnœa, inconsiderable mucous expectoration, pretty good resonance, and nothing unusual discovered by auscultation. Circulation not well distributed to the extremities—pulse eighty, small and feeble—no particular examination made of the heart.—*Abdomen*—tongue slightly covered with a whitish fur; appetite not good, but the stomach retained and digested very well whatever of food was taken; bowels, in a good condition, no pain nor tenderness, upon pressure, in any part of the abdomen.

*Diagnosis* uncertain; most probably chronic bronchitis, consequent upon the cold taken last winter. Directed vegetable diet—cupping over the anterior parts of thorax—brown mixture, and mucilaginous drink. Found him at next visit somewhat relieved of the dyspnœa, but cough still troublesome. Brown mixture continued.

There being no manifest change in two days, he was ordered ung. tart. emetic, to free pulsation on anterior thorax; brown mixture to be continued.

A copious crop of pustules was produced in a day or two, when we discovered great force in the action of the heart, communicating a considerable impulse to the hand, and attended with acute pain upon pressure. Tinct. digital. was added to the brown mixture, and cups were applied to the præcordia.

The pain was mitigated by the cupping, but this great activity in the pulsation of the heart having continued unintermittingly for several days, and the dyspnœa, dry cough, and feeble pulse, which was very small considering the activity of the heart, still continuing, led us to believe that there was contraction of the left ventriculo-aortic valves, notwithstanding we could detect no morbid alteration in the sounds of the heart. We could not only easily account for the symptoms just named, upon the supposition of this lesion of the aortic semi-lunar valves, but we were compelled to suppose this or some affection of the aorta itself, which was not then indicated by any particular symptom. Brown mixture discontinued. Pulv. digital. gr. i. morning, noon, and night.

This was continued several days, when we were called to him early in the morning, and found him labouring under extreme dyspnœa—heart beating violently—pulse much fuller and stronger than we had ever seen it, and had only changed within a few minutes, as we were informed by the house student—surface cold, and covered with a cold perspiration; face livid and turgid with blood—eyes semi-closed, and pupils dilated—jugulars stuffed—total insensibility—brain evidently greatly engorged and oppressed with blood, and apoplexy seeming to be momentarily threatened. Before we reached the house, sinapisms had been applied to the breast and extremities, and an effort made to abstract blood; only two or three ounces could be obtained. A large orifice was immediately opened in the arm, and about thirty ounces of blood soon abstracted, with almost entire relief. His condition just described, together with the effect produced by thus rapidly diminishing the quantity of blood in circulation, confirmed us in the view which we had taken. These paroxysms continued to recur daily, and frequently two or three times a day, and always required the abstraction of blood before relief was obtained. We found that relief was afforded more speedily and with the loss of a smaller quantity of blood, which became desirable from the necessity of its frequent repetition, by opening a jugular. So satisfied was he that blood-letting was his only source of relief, that he implored it as an act of mercy, when a paroxysm was about to come on.

A few days before death, the nature of the affection became apparent from an increased fulness which was observed to make its appearance, extending two or three inches by the side of the left edge of the sternum,—commencing at the top,—from a palpitation which became very obvious at the intercostal spaces of the part, and from his complaining of a "lump which choked him" about the top of the sternum. Death took place on the 8th of July, during one of the paroxysms already described.

*Examination of the Body Nine Hours after Death.*

*Head.* No cerebral symptoms having been observed since opening the jugular had been resorted to, this cavity was not examined.

*Thorax—Lungs.*—Pulmonary veins partially engorged with very black blood—normal in every other respect, save interlobular emphysema which was pretty generally present throughout the lungs. *Heart.*—Right auricle and ventricle contained fibrinous concretions—the auricle containing also a coagulum of black blood—while the ventricle was almost entirely filled with an opaque, firm concretion, which adhered with some firmness to the columnæ carneæ; there was also a dark coagulum in the left auricle, but the left ventricle was empty. No particular lesion of the heart was discoverable, save a slight hypertrophy, without dilatation, of the left ventricle. *Aorta.*—At its arch we found an aneurismal sac, a cylindroid in shape, about two and a half inches in length, and two in diameter. It contained a small opaque coagulum, and probably others, but wishing to preserve it entire we did not lay it open. By the whole of its posterior surface it adhered very firmly, indeed inseparably, to the anterior surface of the trachea, just above its bifurcation. The coats of the sac were entire.

*Abdomen.*—No lesion, of consequence, of any of its viscera.

The heart and aorta, with the adherent trachea, were placed in the Pathological Cabinet of the Louisville Medical Institute, where they may be seen.—*Dr. Bayless' Hospital Reports in Western Journal of Medicine and Surgery.*

